# The pathophysiology of intestinal lipoprotein production

**DOI:** 10.3389/fphys.2015.00061

**Published:** 2015-03-20

**Authors:** Antonina Giammanco, Angelo B. Cefalù, Davide Noto, Maurizio R. Averna

**Affiliations:** Dipartimento Biomedico di Medicina Interna e Specialistica, Università degli Studi di PalermoPalermo, Italy

**Keywords:** chylomicron, triglyceride-rich lipoproteins, assembly, secretion, inherited disorders

## Abstract

Intestinal lipoprotein production is a multistep process, essential for the absorption of dietary fats and fat-soluble vitamins. Chylomicron assembly begins in the endoplasmic reticulum with the formation of primordial, phospholipids-rich particles that are then transported to the Golgi for secretion. Several classes of transporters play a role in the selective uptake and/or export of lipids through the villus enterocytes. Once secreted in the lymph stream, triglyceride-rich lipoproteins (TRLs) are metabolized by Lipoprotein lipase (LPL), which catalyzes the hydrolysis of triacylglycerols of very low density lipoproteins (VLDLs) and chylomicrons, thereby delivering free fatty acids to various tissues. Genetic mutations in the genes codifying for these proteins are responsible of different inherited disorders affecting chylomicron metabolism. This review focuses on the molecular pathways that modulate the uptake and the transport of lipoproteins of intestinal origin and it will highlight recent findings on TRLs assembly.

## Introduction

Dietary fats are taken up by enterocytes of the small intestine and packaged into chylomicrons. Chylomicrons (CMs) are triglyceride-rich lipoproteins (TRLs) with a central lipid core and a layer of phospholipids (6–12%), free cholesterol (1–3%), and apolipoproteins (1–2%) and play an essential role in the transport of triglycerides and fat-soluble vitamins (Hamilton, 1972). Hepatocytes, in turn, secrete TRLs as very low density lipoprotein (VLDL)particles (Hamilton, 1972). Both CMs and VLDLs are hydrolyzed mainly by lipoprotein lipase (LPL) in the circulation and processed into CM remnants and low density lipoprotein (LDL) respectively. In this review we will focus on the physiological mechanisms, pathways and genes that modulate the uptake and the transport of lipoproteins of intestinal origin. We will first discuss the synthesis and maturation steps of the primordial lipoprotein particles of intestinal origin and the processes of secretion in the circulation. Afterward, we will briefly focus on the extra-intestinal fate of CMs and we will give an overview on the inherited disorders affecting CMs metabolism. Finally we will highlight recent findings on TRLs assembly.

## Intestinal lipid absorption: an overview

Growing bodies of evidences indicate, both in humans and animal models, that the small intestine is not only involved in the absorption of dietary lipids but actively regulates the production and secretion of CMs (Abumrad et al., 1993; Cartwright et al., 2000; Mansbach and Gorelick, 2007; Khosrow and Lewis, 2008). The process of dietary lipid absorption is traditionally divided into three components: (a) uptake into the enterocyte, (b) intracellular processing, and (c) transport into the circulation (Mansbach and Gorelick, 2007; Khosrow and Lewis, 2008; Abumrad and Davidson, 2012). Pancreatic lipase makes the first step possible through the hydrolysis of dietary fats, mostly triacylglycerols (TAG), within the lumen of the small intestine. Fatty acids (FA) and sn-2-monoacylglycerol (MAG) are the results of this enzymatic breakdown (Mansbach and Gorelick, 2007). Hydrolysis products are then transported across the apical brush border membrane of the enterocyte by cluster determinant 36 (CD 36) (Abumrad and Davidson, 2012). The FA are then bound by FA binding proteins (FABPs) and targeted to microsomal compartments for re-esterification to triglycerides (Khosrow and Lewis, 2008). De-novo lipogenesis represents another valid source of triglycerides useful for lipidation and this process is hormone-dependent (Khosrow and Lewis, 2008).

CM assembly is a complex process that needs the activity of microsomal triglyceride transfer protein (MTP) to cotranslationally incorporate apoB-48 into a phospholipids-rich, dense, primordial chylomicron particle (prechylomicron) (Kumar and Mansbach, 1999; Hussain, 2000; Levy et al., 2002). Then, prechylomicrons are included in a unique transport vesicle, the prechylomicron transport vesicle (PCTV), which is budded off the endoplasmic reticulum (ER) membrane and transported to the Golgi (Kumar and Mansbach, 1999; Hussain, 2000). Once into the Golgi compartment several chylomicrons fuse into another transport vesicle and are transported to the basolateral membrane for secretion in the circulation (Hussain, 2000; Jones et al., 2003).

Two different models have been proposed for CMs assembly (Cartwright et al., 2000; Hussain, 2000). According to Hussain, the assembly of small nascent lipid poor CM particles and buoyant triglyceride-rich chylomicrons progress through independent pathways (Hussain, 2000).

On the other hand the so called “core expansion” model, proposes that primordial chylomicrons and triglyceride-rich lipid droplets of various sizes join together to form lipoproteins of different size (Cartwright et al., 2000).

In the next sections we will focus on recent advancing in understanding the process of intestinal assembly and trafficking of CMs.

## Major pathways and genes involved in chylomicrons metabolism

Small intestine enterocytes absorb cholesterol and FA both through passive diffusion and protein-facilitated FA transfer (Abumrad and Davidson, 2012). Over the years, several classes of long chain fatty acids (LCFA) transporters have been shown to be involved in the selective uptake and/or export of dietary lipids to the sub-apical domain of the villus enterocyte (Abumrad and Davidson, 2012). CD 36, originally identified as a receptor for oxidized low-density lipoproteins (Endemann et al., 1993), is a 75–88 kDa, 472 aminoacid glycosilated transmembrane protein, which actually show a wide ligand specificity for LCFA, different lipoproteins, glycosylated proteins, thrombospondin-1 and other molecules (Silverstein and Febbraio, 2009). CD 36 is ubiquitously expressed and major sites of synthesis are heart, skeletal muscle, adipose tissue, intestine (Abumrad et al., 1993), and the capillary endothelium (Greenwalt et al., 1995). Several *in vitro* and *in vivo* studies have demonstrated that CD 36 facilitates the LCFA tissue uptake (Coburn et al., 2000; Hajri et al., 2002) and it plays a relevant role in the pathogenesis of metabolic disorders and atherogenesis (Abumrad et al., 1993; Watanabe et al., 1998; Hirano et al., 2003; Ma et al., 2004; Yamashita et al., 2007; Love-Gregory et al., 2011).

The uptake of FA both in the intestine and liver is also mediated by fatty acid binding proteins (FABPs). FABPs are small cytosolic proteins (14 kDa) belonging to a multigene family expressed both in the intestine (Intestinal-FABP, IFABP or FABP2) and in the liver (liver-FABP, LFABP or FABP1) (Abumrad et al., 1993). IFABP is able to bind one FA molecule while LFABP binds two FA molecules in addition to a wide range of other lipids (Hamilton, 2004; Storch and Thumser, 2010).

Intestinal triglyceride-rich lipoprotein assembly needs two major players, both of which must be available simultaneously for this process to proceed to completion: (a) apolipoprotein B (apoB), which is the acceptor for the neutral lipid donor and (b) microsomal triglyceride transfer protein (MTP) (Chen and Davidson, 2012). ApoB is an essential structural component of the surface of lipoprotein particles. TRLs cannot be assembled without apoB being present. In humans, TRLs are assembled in liver and intestine in a tissue-specific manner. In the liver full-length apoB (apoB-100) is assembled into VLDL (Davidson and Shelness, 2000). In human enterocytes, a post-transcriptional editing of the apoB mRNA transcript gives rise exclusively to a truncated form of apoB (apoB-48) (Davidson and Shelness, 2000) for the assembly of CMs (Hussain, 2000). CMs are larger particles than hepatic VLDLs and beside apoB they also contain multiple copies of smaller apolipoproteins including apoA-I, apoA-IV and apoC-III. These apolipoproteins are important for VLDL and/or chylomicrons assembly but not essential as apoB (Hussain, 2000; Hussain et al., 2005).

The nascent ApoB is translocated across the ER membrane where MTP interacts with the apoB N-terminal domain (Liang et al., 2000). This interaction promotes the transfer of lipids from the ER membrane to the nascent apoB leading to the formation of a primordial lipoprotein particle (Liang et al., 2000). When lipid availability is limited or MTP function impaired, the nascent apoB becomes misfolded and undergoes ubiquitination and degradation through the proteasomal pathway (Liang et al., 2000). This step may occur either outside the ER or within the ER lumen (Fisher et al., 2001). MTP also directs the assembly of apoB-free triglyceride-rich particles within the adjacent smooth ER (SER) (Mansbach and Gorelick, 2007). During intracellular lipoprotein assembly, the nascent lipoprotein particle joins with SER TG-rich lipid droplets, resulting in prechylomicron formation (Mansbach and Gorelick, 2007). Once this process is completed prechylomicrons are enriched of vesicular transport proteins, including coatomer proteins II (COPII) to be incorporated into a vesicular complex through ER membrane budding ready to fuse with Golgi membranes apparatus (Jones et al., 2003; Mansbach and Gorelick, 2007). Vesicles containing nascent CMs are then secreted into the pericellular spaces adjacent to lymphatic fenestrae (Mansbach and Gorelick, 2007). The COPII machinery has been shown to have a critical role in transporting TRL particles from the ER to the Golgi (Barlowe et al., 1994; Ikonen, 2008). Among the protein components of COPII machinery, Sar1b, a member of the Sar1/Arf family of small GTPase, is an essential player in the last step of assembly of this vesicular transport complex (Shoulders et al., 2004). Chylomicron-retention disease (CMRD) due to mutations in the SAR1B gene, encoding for Sar1b, is an example of failure in the transport of prechylomicrons through the secretory pathway even though the ER assembly process takes place correctly (Cefalù et al., 2010; Shoulders et al., 2014). Therefore, prechylomicrons accumulate in the membrane-bound compartments of enterocytes. Notably mutations of the SAR1B gene that are associated with CMRD do not impair VLDL packaging and secretion (Jones et al., 2003; Shoulders et al., 2004). This observation warrants further evaluations to better understand the mechanisms regulating intestinal lipid assembly and secretion.

## Biogenesis of chylomicrons: a multistep process

### Chylomicron formation within the ER lumen

**Figure 1:**
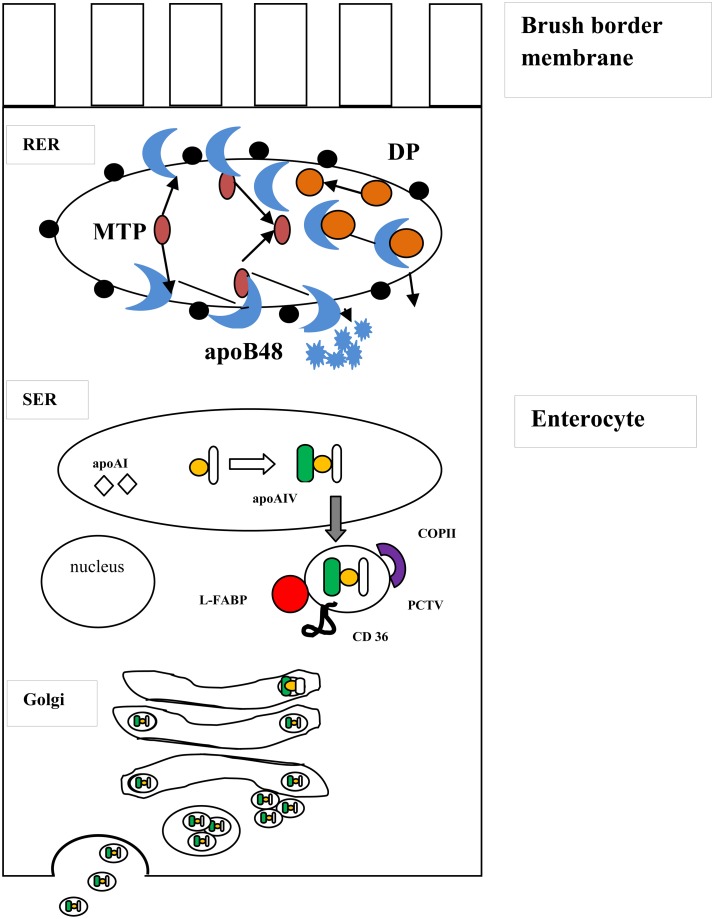
**Biogenesis of chylomicrons**. The formation of chylomicrons occurs in a two-step process within the ER lumen. In the first step, newly synthesized apolipoprotein B48 (in blue) is chaperoned by the luminal protein, microsomal triglyceride transfer protein (MTP, in purple) into the rough endoplasmic reticulum (RER). The apoB then follows two destinies: it can form stable complexes with dense particles (DP, in orange) containing mostly phospholipids, cholesterol and small amounts of triacylglycerol (above) or is rapidly degraded in the absence of association with lipids (below). In the smooth endoplasmic reticulum (SER), the triacylglycerol and cholesterol esters (in yellow) are carried from their site of synthesis on the ER membrane to an enlarging particle through MTP and is incorporated to apolipoprotein A-IV (apoA-IV, in green) to form a large particle, which buds from the SER surrounded by a membrane, resulting in prechylomicron transport vesicle (PCTV) formation. Other proteins, including L-FABP (in red) and CD 36 (in black), also participate in PCTV formation. After fusion with key vesicular transport proteins, such COPII (in violet), the prechylomicrons are incorporated into a vesicular complex that translocate to and fuse with the Golgi complex. Here, apoA-I arrives in different transport vesicles than PCTV and attaches to the chylomicrons to form a mature chylomicron containing apoA-I, apo A-IV and apo B48 (not shown). The mature chylomicrons exit the Golgi complex in large transport vesicles that fuse with the basolateral membrane and are secreted.

CMs are synthesized through a multistep process that begins in the endoplasmatic reticulum (Figure 1). In the first step a high-density particle consisting of apoB-48, phospholipids, apoA-IV, cholesterol, and small amounts of TAG is formed to give rise nascent chylomicron particles. Then, these particles join with a large apoB-free TAG-cholesterol ester mass to form a prechylomicron particle, which is transferred to the *cis*-Golgi (Yamaguchi et al., 2006; Mansbach and Gorelick, 2007). In the rough endoplasmic reticulum (RER), newly synthesized apoB-48 interacts with the luminal MTP. ApoB-48 then may follow two destinies: (a) it may form tight complexes with lipid rich particles containing mostly phospholipids, cholesterol and small amounts of triacylglycerol or (b) it may be rapidly degraded if association with lipids does not occur. In the SER, triacylglycerol and cholesterol esters are transported by MTP from their site of synthesis on the ER membrane to an enlarging particle stabilized by a phospholipid-cholesterol monolayer and merged with apoA-IV to form a large light particle (LP). This particle buds from the SER surrounded by a membrane. It has been reported that this step is mediated by L-FABP, but not COPII proteins and it also requires ATP (Yamaguchi et al., 2006; Mansbach and Gorelick, 2007). If TAG-rich particles lack apoB-48 are unable to leave the ER suggesting that the apoB-48 on the surface of the TAG-rich particle indicates that the prechylomicron is ready to be exported (Ojakian et al., 2006; Mansbach and Gorelick, 2007).

### Export of pre-chylomicrons from the ER

After the pre-chylomicrons are synthesized, these intracellular particles leave the ER and are vehicled through the secretory pathway for further modification (Abumrad and Davidson, 2012).

Once prechylomicrons are formed in the ER they need to translocate to and fuse with the Golgi complex. There are some important distinctions between the chylomicron transport system and the transport of other newly synthesized proteins. In fact the very large size of prechylomicrons requires a specific transporter; PCTVs are large enough to carry prechylomicrons and are able to fuse with the *cis*-Golgi (Mansbach and Gorelick, 2007). Furthermore, PCTVs are unique compared to other protein vesicles because they enclose the v-soluble N-ethylmaleimide-sensitive factor attachment protein receptor (v-SNARE) and the vesicle associated membrane protein 7 (VAMP-7) (Siddiqi et al., 2003, 2010a; Mansbach and Gorelick, 2007). Several studies suggest that the COPII proteins are able to select protein cargo to be included into the vesicle, deform the ER membrane and break up the budding vesicle from the ER membrane to form the nascent protein transport vesicle. These events take place at specific ER exit sites marked by secretory proteins (Sec16 and Sec23) (Mansbach and Gorelick, 2007). These transport vesicles protect their cargo from cytosolic proteases by providing a closed space and contain on their surface information that enables them to be targeted to the *cis*-Golgi (Mansbach and Gorelick, 2007).

The COPII machinery plays a unique role in vesicular budding and trafficking of ER cargo destined for secretion through the Golgi apparatus (Mansbach and Siddiqi, 2010). Studies in rat enterocytes demonstrated that the sequential interaction of heterodimeric COPII proteins (Sec 23/24 and Sec 13/31) is required for prechylomicrons budding from the ER to the Golgi apparatus (Siddiqi et al., 2010a,b). Data from animal model (adult rat) have suggested that budding of the PCTV is the rate-limiting step in lipid absorption (Siddiqi et al., 2006). Neeli et al. (2007) have recently indicated that L-FABP can select cargo and bud the PCTV from the ER membrane. Studies on intestinal extracts suggest that ER budding and formation of the PTCVs do not require COPII proteins while trafficking and fusion with the *cis*-Golgi is dependent on COPII proteins Sar1b and Sec23/24 (Siddiqi et al., 2010a) as well as a soluble N-ethylmaleimide-sensitive factor attachment protein receptor (SNARE), fusion complex composed of vesicle-associated membrane protein 7 (VAMP7), synthaxin 5, Bet1, and vti1a (Black, 2007). PCTV budding is impaired in intestinal extracts prepared from *CD36*−/− mice, suggesting a role for this multifunctional receptor (Siddiqi et al., 2003). Moreover, studies in *L-Fabp*−/− and in *CD 36*−/− mice have also shown that the kinetics of intestinal TG secretion is significantly slower suggesting an important functional regulation of PCTV formation arbitrated by L-FABP and CD 36 (Drover et al., 2005, 2008; Newberry et al., 2006). On the other hand, studies in a rat hepatoma cell model demonstrated that a functional COPII complex is required for ER budding of the nascent apoB100-VLDL particle (Gusarova et al., 2003). Studies on intestinal PCTV have been suggested a potential mechanism of the CM-independent secretion of apoprotein A-I (ApoA-I), another apolipoprotein involved in CM assembly (Mansbach and Gorelick, 2007). Indeed ApoA-I secretion is not combined with the CM secretion (Mansbach and Gorelick, 2007) probably because PCTV are lacking of apoA-I (Siddiqi et al., 2003) and the transport vesicle that exports apoA-I from the ER to the Golgi is different and likely to be the COPII-dependent protein transport vesicle. Therefore, at the Golgi, apoA-I arrives in different transport vesicles than the PCTV (Siddiqi et al., 2003). Once into the Golgi apparatus, apoA-I binds to the prechylomicron to form a mature chylomicron containing apoA-I, apoA-IV and apoB-48 (Mansbach and Gorelick, 2007). The mature chylomicrons leave the Golgi complex in large transport vesicles that join with the basolateral membrane and are finally secreted (Mansbach and Siddiqi, 2010).

### Chylomicron secretion

CMs output into the circulation correlates with the amount of dietary phosphatidylcholine (PC) in the intestinal lumen, the amount of fat in the diet, the expression of apoA-IV by the enterocytes and the hydratation state of the mucosa (Voshol et al., 2000).

High fat diet increases the transport of biliary PC and apoA-IV plays a role in the induction of the CM-TAG secretion mainly by increasing the size of the CMs (Lu et al., 2006; Black, 2007).

In addition the ability to export CMs into the blood stream depends of the hydration state of the intestine.

ApoA-IV is a lipid-binding protein that is synthesized by enterocytes in response to lipid absorption (Green et al., 1980; Apfelbaum et al., 1987; Elshourbagy et al., 1987) and it is secreted in association with primordial chylomicrons into mesenteric lymph (Kalogeris et al., 1996; Kumar and Mansbach, 1999); once CMs are secreted and undergo lipolysis, a significant fraction of the apoA-IV dissociates because of the narrowing of CMs surface and then is transferred to HDL and lipoprotein-free fractions (Tso et al., 1984; Ohta et al., 1985; Hayashi et al., 1990). Intestinal lipid absorption, as well as CM secretion, regulates ApoA-IV expression (Zannis et al., 1980; Weinberg et al., 1990; Kalogeris et al., 1997). Therefore, the presence of apoA-IV in the peripheral circulation is dependent on the intestinal formation and secretion of lipid. ApoA-IV also cooperates in several physiological functions as an antioxidant (Kalogeris et al., 1998), anti-inflammatory factor (Vowinkel et al., 2004), anti-atherosclerotic factor (Duverger et al., 1996; Cohen et al., 1997), a mediator of reverse-cholesterol transport (Dvorin et al., 1986; Stein et al., 1986), and acute satiety factor (Fujimoto et al., 1992; Tso et al., 2001). All of these important functions of apoA-IV may be redundant roles since other apolipoproteins are involved in such processes (Green et al., 1980; Apfelbaum et al., 1987; Elshourbagy et al., 1987).

ApoA-IV neither acts to stabilize the CM-TAG, nor it is necessary for the packaging of TAG into CMs but it is mainly involved in secretion and further metabolism in the circulation (Kohan et al., 2012, 2013; Wang et al., 2012). Moreover the lack of apoA-IV delays the clearance of CMs (Kohan et al., 2012).

Recently it has been shown that apoA-IV plays an important role in mediating glucose homeostasis by stimulating insulin secretion and that the loss of apoA-IV significantly impairs glucose homeostasis and peripheral lipid clearance. ApoA-IV KO mice display defective insulin release from pancreatic islets and impaired glucose tolerance compared to wild type (WT) mice, particularly on high fat diet (HFD) (Wang et al., 2012). Administration of exogenous apoA-IV in apoA-IV KO mice improves glucose tolerance improving insulin secretion rates (Wang et al., 2012). A recent report has shown that in patients undergoing gastric bypass surgery plasma levels of apoA-IV increases after surgery and correlates with an improved glucose tolerance (Culnan et al., 2009). ApoA-IV appears to be an endogenous regulator of insulin secretion with tight interactions with other components of the enteroinsular axis.

ApoA-IV is post-translationally regulated by the glucagon-like peptide-1 (GLP-1), a gut incretin that modulates insulin secretion in response to carbohydrates and FA intake (Drucker, 2001; Hein et al., 2013). GLP-1 acts by stimulating insulin secretion and decreasing the availability of apo B-48 and TG for assembly and secretion of CMs (Hsieh et al., 2010). Moreover the administration of exogenous recombinant GLP-1 (rGLP-1) in diabetic rats inhibits apoA-IV secretion and TG absorption (Drucker, 2001; Qin et al., 2005).

Recent evidences have also emphasized the crucial role of another intestinal peptide, the glucagon-like peptide-2 (GLP-2), in the intestinal lipid homeostasis (Estall and Drucker, 2006; Hsieh et al., 2008; Hein et al., 2013; Dash et al., 2014). GLP-2 is secreted together with GLP-1, but it exerts opposite effect on CMs assembly and secretion and specifically it enhances the secretion of CMs from enterocytes. (Estall and Drucker, 2006; Meier et al., 2006; Hsieh et al., 2010; Dash et al., 2014). When GLP-1 and GLP-2 are co-infused intravenously in normal subjects for a short time (30 min) lipid absorption is increased suggesting a predominat GLP-2 action (Hein et al., 2013). However, under prolonged (120 min) co-infusion, the action of GLP-1 prevails by decreasing post-prandial CMs assembly and secretion (Hein et al., 2013). It's notheworthy that GLP-1 is rapidly degraded by the enzyme dipeptidyl peptidase-4 (DPP-4) (Hein et al., 2013). All together these findings suggest that promoting GLP-1 activity, either by agonist-based therapies or DPP-4 inhibitors, may be helpful to manage dyslipidemia in insulin-resistant/diabetic conditions (Hsieh et al., 2010; Xiao et al., 2012, 2014; Hein et al., 2013; Matikainen and Taskinen, 2013). On the other hand, antagonizing GLP-2 may be a good strategy to decrease the post-prandial secretion of CMs (Dash et al., 2014). All together these data warrant further investigations on the role of apoA-IV, GLP-1, and GLP-2 as modulators of lipid and glucose homeostasis.

## Extra-intestinal mechanisms of triglyceride-rich lipoproteins metabolism

Once chylomicrons are secreted and transported *via* the intestinal lymphatic system they enter the blood stream where they are metabolized by Lipoprotein lipase (LPL), which plays a key role in the hydrolysis of triacylglycerols of VLDLs and CMs. LPL is synthesized in parenchymal cells of various organs, including adipose and skeletal muscle, and it is secreted and transported to the luminal surface of vascular endothelium where hydrolysis occurs. Active LPL is a homodimer that is bound to heparan sulfate chains on the surface of endothelial cells (Cheng et al., 1981; Cupp et al., 1987). LPL requires apolipoprotein C-II (APOC-II) as a cofactor for efficient lipolysis of TRLs in the circulation (Wilson, 1990). The lack of ApoC-II or defects in its structure severely impair LPL-mediated lipolysis and consequently TGs accumulate in plasma (Di Filippo et al., 2014). Recently, three new proteins have been identified as essential players for proper LPL function: apoA-V, lipase maturation factor 1 (LMF1) and glycosylphosphatidylinositol-anchored HDL binding protein 1 (GPIHBP1). The primary known function of apoA-V is to act as cofactor and stimulate LPL and plasma clearance of TRLs (Gonzales et al., 2013) LMF1 has been shown to be essential for the maturation of both LPL and hepatic lipase (HL) to their full functional forms (Peterfy et al., 2007). GPIHBP1 has been identified as the endothelial protein that facilitates LPL trafficking toward the endothelial cell surface and provides a platform for TG lipolysis (Beigneux et al., 2007; Davies et al., 2010).

## Inherited disorders affecting chylomicron metabolism

As previously discussed MTP is an intracellular lipid-transfer protein essential for the assembly and secretion of VLDL particles and CMs by initiating the incorporation of lipids into ApoB and by acting as a chaperone to assist in the proper folding of the Apo B protein (Kane, 1989; Wetterau et al., 1997). The role of MTP in lipid transport and metabolism was revealed by studies demonstrating that a genetic defect in MTP gene causes abetalipoproteinemia, a condition characterized by lack of production of ApoB containing lipoproteins (CMs and VLDL).

Abetalipoproteinemia (ABL; OMIM 200100) is a rare autosomal recessive disease due to mutations in both alleles of the MTP gene; affected patients exhibit nearly undetectable levels of ApoB and very low plasma cholesterol levels. Due to the impairment of lipoprotein assembly, patients develop fat malabsorption, steatorrhea and fat accumulation in enterocytes and hepatocytes (Kane, 1989; Wetterau et al., 1997). The steatorrhea is usually minimized as subjects learn to avoid fat rich diets at an early age. Subjects also suffer from multiple vitamin deficiencies (E, A, K, and D) because these fat soluble vitamins require the intestinal TRLs for normal absorption and transport. If untreated, affected subjects will develop various neurological disturbances including spinal-cerebellar degeneration, peripheral neuropathies and retinitis pigmentosa. These conditions can be prevented by supplementation of fat soluble vitamin early in life (Haghpassand et al., 1996; Wetterau et al., 1997; Hussain et al., 2012).

The role of molecular inactivating defects of MTP as cause of ABL suggested that inhibition of MTP could be a novel pharmacological target to lower plasma lipid levels.

Recently a phase 3 trial with lomitapide, a pharmacological inhibitor of MTP has been successfully used as adjunctive treatment in homozygous familial hypercholesterolemia (HoFH) patients (Cuchel et al., 2013; Golberg, 2013).

Homozygous hypobetalipoproteinemia (Ho-HBL; OMIM 107730) is another extremely rare inherited disorder characterized by improper packaging and secretion of apoB-containing lipoproteins due to mutations in both alleles of the APOB gene (Schonfeld, 2003; Tarugi and Averna, 2011). The majority of them are either carriers of homozygous or compound heterozygous mutations in APOB gene leading to apoB truncations or amino acid substitutions (Lee and Hegele, 2014; Cefalù et al., 2015). The clinical manifestations of patients with molecularly defined Ho-FHBL are variable ranging from lack of symptoms to the presence of clinical features overlapping with those of ABL (Lee and Hegele, 2014; Cefalù et al., 2015).

When truncated apoBs species are smaller than apoB-48, the apoB form produced by the intestine and incorporated into chylomicrons, these short abnormal apoBs lose the capacity to bind lipids and form chylomicrons that are secreted from the enterocytes into the intestinal lymph (Tarugi and Averna, 2011; Lee and Hegele, 2014; Cefalù et al., 2015).

Chylomicron retention disease (CMRD) or Anderson's Disease (OMIM #607689) is a rare, autosomic recessive disorder usually diagnosed in infants presenting with chronic diarrhea, failure to thrive, hypocholesterolemia and low fat-soluble vitamin levels (Anderson et al., 1961; Lee and Hegele, 2014; Cefalù et al., 2015). The specific molecular defect was identified in 2003 and consists of mutations in the SAR1B gene which encodes for intracellular Sar1b protein (Strich et al., 1993; Shoulders et al., 2004; Hussain et al., 2005; Cefalù et al., 2010). This protein is involved in the transport of chylomicron through the secretory pathway (see Section Chylomicron Secretion). The enterocytes of jejunal mucosa of these patients fail to secrete CMs in the lymph and are consequently overloaded with lipid droplets heterogeneous in size and distribution (Strich et al., 1993). The mucosal surface of the small intestine, as observed by endoscopy, is covered with a whitish layer (“a white stippling-like hoar frosting” or gele'e blanche). Bloating of the abdomen, osteomalacia, and rickets have been observed in several cases. Fatty liver has been described without any evolution to more severe forms of hepatic disease (Charcosset et al., 2008). CMRD is clinically distinguishable from ABL and Ho-HBL by the absence of acanthocytosis, retinitis pigmentosa, and severe neurological symptoms and the detection of circulating apoB-100-containing lipoproteins.

HTG is a hallmark of both Fredrickson type 1 and type 5 hyperlipidemias. Type 5 hyperlipidemia encompasses a mixed hyperlipidemic phenotype including elevated levels of both CM and VLDL remnants particles (Xiong et al., 1991; Brunzell and Fujimoto, 1995; Berriot-Varoqueaux, 2004). The clinical syndrome, referred to as Type I hyperlipoproteinemia, also known as familial chylomicronemia, is characterized by massive hypertriglyceridemia, abdominal pain, pancreatitis, eruptive xanthomas and hepatosplenomegaly occurring at a frequency of about one in a million in general population (Xiong et al., 1991; Brunzell and Fujimoto, 1995; Berriot-Varoqueaux, 2004).

LPL and APOC-II gene mutations (Breckenridge et al., 1978; Auwerx et al., 1989; Brunzell and Austin, 1989) together with mutations occurring in the APOA-V, LMF1 and GPIHBP1 are responsible for monogenic forms of familial chylomicronemia (Monsalve et al., 1990; Marcais et al., 2005; Priore et al., 2005; Beigneux et al., 2009; Johansen et al., 2011). Affected individuals are either homozygous or compound heterozygous for loss-of-function mutations in these genes involved in the regulation of catabolism of TRLs. Affected patients show substantially increased fasting concentrations of CMs, but usually do not develop premature atherosclerosis, probably because of size exclusion that limits the ability of CMs to traverse the vascular endothelial barrier (Xiong et al., 1991; Brunzell and Fujimoto, 1995; Johansen et al., 2011). Lipoprotein lipase deficiency (LPLD) can be biochemically diagnosed by measurement of enzymatic activity of LPL in post-heparin plasma or in adipose tissue (Wilson et al., 1990; Hegele et al., 2013). At present, the gold standard for diagnosis of Familial Chylomicronemia relies on DNA sequencing for detection of pathogenic mutations in LPL and/or other candidate genes (Johansen et al., 2011).

## Emerging role of sortilins in triglyceride rich lipoprotein assembly

A number of new proteins have been recently identified as important players in TRLs assembly.

Sortilin-1 is one of five members of a domain receptor family of the *trans*-Golgi network and early endosomes known as vacuolar protein sorting 10 proteins (Vps10p) (Charcosset et al., 2008). It shows a wide range of ligands and it has been demonstrated to play a role in the clearance of apoB-containing lipoproteins (Strong and Rader, 2012). Sortilin serves as both a chaperone and degrader of apoB-containing lipoproteins in a concentration-dependent manner. Sortilin has been recently shown to play a role in liver VLDL secretion. Specifically, low levels of sortilin may be required for efficient VLDL export; on the other hand higher expression of sortilin promotes the degradation of presecretory VLDL (Wilson et al., 1990).

**Figure 2:**
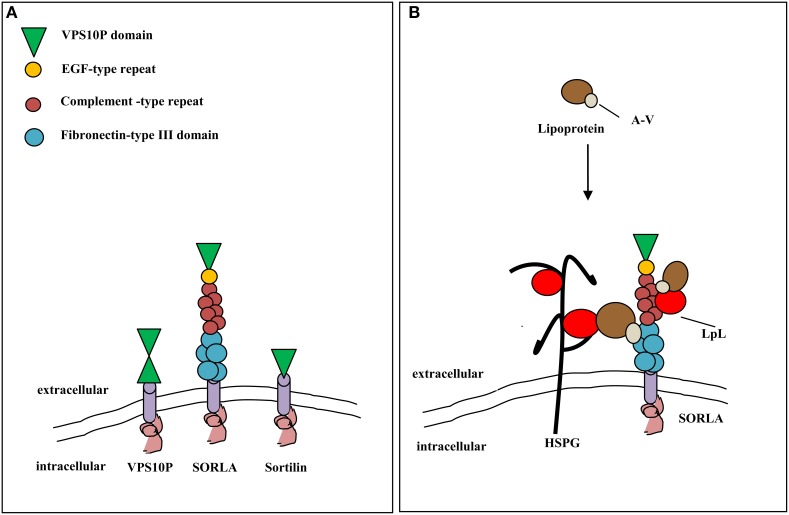
**Vacuolar protein sorting 10 protein domain receptor/sortilin and trygliceride-rich lipoprotein metabolism. (A)** Structural organization of VPS10P domain receptors from yeast (VPS10P) and mammals (sortilin, SORLA). The extracellular domains of the receptors are either composed of one or two VPS10P domains and may carry additional modules involved in protein-protein interaction. EGF, epidermal growth factor, SORLA, sorting protein-related receptor with complement-type repeats. **(B)** SORLA may promote lipolysis of trygliceride-rich lipoproteins through interaction with apolipoprotein A-V and lipoprotein lipase (right side). Alternatively, lipoproteins can bind to SORLA through cofactor apoA-V (left side) and LPL bound to heparin sulfate proteoglycans (HSPG) on the cell surface.

Recently, it has been proposed that ApoA-V acts as cofactor for LPL through the binding of the complex of sortilin and/or other sortilins (as sorting protein-related receptor containing LDLR class A repeats—SORLA/LR11) on the heparan sulfate proteoglycans (HSPG) of endothelial cell surfaces (Figure 2; Babirak et al., 1989; Pennacchio et al., 2001; Van der Vliet et al., 2001; Mendoza-Barbera et al., 2013).

SORLA is a multifunctional receptor expressed in macrophages and vascular smooth muscle cells (SMC) (Van der Vliet et al., 2001). It may act as proatherogenic factor by promoting intimal SMC migration and by regulating apoA-V dependent activation of LPL thus modulating triglyceride plasma levels (Mendoza-Barbera et al., 2013). The expression of SORLA gene is also modulated by factors inducing ER stress which is emerging as an important modulator of atherosclerosis and TRLs secretion (Willnow et al., 2011; Klingenberg et al., 2013; Mendoza-Barbera et al., 2013).

Moreover, recent data from genome-wide association studies (GWAS) have underlined the role of sortilin-1 (SORT1) as lipid regulatory gene in atherosclerotic cardiovascular disease (Teslovich et al., 2010; Strong et al., 2014). In this context an altered trafficking pathways may represent a major risk factor for dyslipidemia and atherosclerosis in the human population (Strong et al., 2014).

## Conclusions

Intestinal lipoprotein production is a complex and multi-step process that involves several and regulated pathways, involving several genes. Recent insights in the understanding of the intestinal and extra-intestinal mechanism of TRLs and their link to atherosclerosis may hopefully facilitate the development of targeted therapeutic approaches to the management of inherited disorders affecting the triglyceride-rich lipoproteins metabolism.

### Conflict of interest statement

The authors declare that the research was conducted in the absence of any commercial or financial relationships that could be construed as a potential conflict of interest.
